# Segmental Dilatation of Ileum Associated with Anterior Thoracolumbar Meningomyelocele and Bilateral Undescended Testes

**Published:** 2015-01-01

**Authors:** Prince Raj, Yogesh Kumar Sarin

**Affiliations:** Pediatric Surgery, Maulana Azad Medical College, and associated Lok Nayak Hospital, New Delhi.

**Keywords:** Segmental dilatation of ileum, Anterior meningomyelocele, Undescended testes

## Abstract

Segmental dilatation of ileum (SDI) is a rare clinical entity and so is anterior thoracic meningomyelocele (AMC). There has been no reported association between these two clinical entities. We hereby report a very rare presentation of these two in a 4 year old boy who presented with swelling in the right lower abdomen. Preoperative diagnoses were partial cecal volvulus and duplication cyst. At operation, SDI along with AMC was found.

## CASE REPORT

A 4-year old boy presented with swelling in the right lower abdomen for the last 3½ years. Swelling was not associated with pain and gradually increased in size as the child grew. Mother gave history of gurgling sound on pressing the swelling. On general examination, the child was pale. There was a non-tender abdominal distention in right iliac region. Testes were bilaterally impalpable. Child was admitted twice for sub-acute intestinal obstruction and was managed conservatively.

Ultrasound revealed a cystic mass lesion in right iliac fossa extending to right flank with internal calcification and was reported as neuroblastoma. USG was repeated which reported dilated bowel loops. MRI was done and revealed large distended bowel loop in right iliac fossa measuring 6.5 cms in diameter with residual food particles and air-fluid levels, suggestive of partial cecal volvulus. There was also a defect seen anteriorly at the level of D12-L1 with prolapsed cerebrospinal fluid filled pouch with few neural components in the abdominal cavity (Fig.1). Both the testes were seen in abdominal cavity. In view of partial cecal volvulus, barium meal study was done, which showed small bowel malrotation with large cavity communicating with ileal loop, suggestive of ileal duplication cyst.

**Figure:**
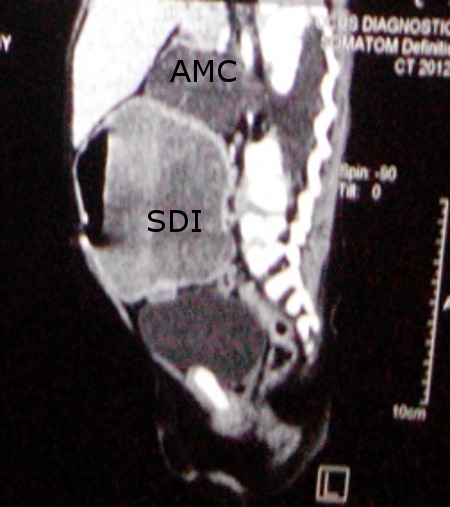
Figure 1: MRI showing SDI and AMC.

At surgery, a large segmental dilatation of terminal ileum (15x10 cms) with Meckel`s diverticulum was found. (Fig.2) Resection of the dilated segment with end-to-end ileo-ileal anastomosis along with excision and repair of AMC were done. The biopsy showed normal bowel musculature with mild mucosal edema, submucosal vascular congestion, serositis, and presence of normal ganglion cells in both the plexi. A month later bilateral Fowler-Stephens stage 1 was done laparoscopically for intra-abdominal testes, with the plan of second stage after 6 months.

**Figure:**
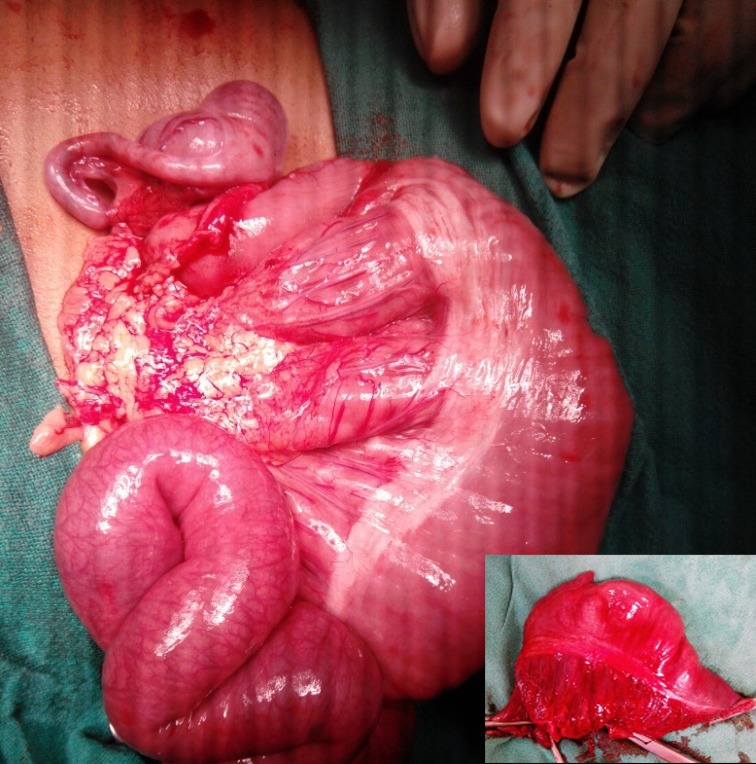
Figure 2: SDI, inset showing resected SDI with Meckel’s diverticulum.

## DISCUSSION

Clinically, the child with SDI may present with nonspecific symptoms as pain abdomen or it may show signs of bowel obstruction, anemia and gastrointestinal bleeding.[1-6] Segmental dilatation of bowel has been associated with omphalocele, Meckel’s diverticulum and malrotation of the gut.[5-7] Our case demonstrates a very rare presentation of SDI associated with AMC, Meckel’s diverticulum, malrotation of the gut and bilateral intra-abdominal testes, which has not been reported in the literature. The cause for this association with AMC can only be speculated. AMC itself is a very rare entity with a few reported cases. The thoraco-lumbar AMC is extremely rare. It could either be a chance association or may have a common embryological basis as complete closure of neural tube occurs by 4 weeks of gestation and at the same time, the foregut, midgut and hindgut start elongating. The presence of the AMC and dilated ileum may have prevented the descent of bilateral testes. Our case also highlights the fact that it is difficult to diagnose SDI based on radiological investigations as none of the investigations done helped in clinching the diagnosis. A very high index of suspicion has to be kept for diagnosing SDI.

## Footnotes

**Source of Support:** Nil

**Conflict of Interest:** None declared

